# Allelopathy of Knotweeds as Invasive Plants

**DOI:** 10.3390/plants11010003

**Published:** 2021-12-21

**Authors:** Hisashi Kato-Noguchi

**Affiliations:** Department of Applied Biological Science, Faculty of Agriculture, Kagawa University, Miki 761-0795, Kagawa, Japan; kato.hisashi@kagawa-u.ac.jp

**Keywords:** allelochemical, decomposition, exudation, invasive plant, mycorrhizal colonization, monospecies stand, phytotoxicity

## Abstract

Perennial herbaceous *Fallopia* is native to East Asia, and was introduced to Europe and North America in the 19th century as an ornamental plant. *Fallopia* has been spreading quickly and has naturalized in many countries. It is listed in the world’s 100 worst alien species. *Fallopia* often forms dense monospecies stands through the interruption of the regeneration process of indigenous plant species. Allelopathy of Japanese knotweed (*Fallopia japonica*), giant knotweed (*Fallopia sachalinensis*), and Bohemian knotweed (*Fallopia* x *bohemica*) has been reported to play an essential role in its invasion. The exudate from their roots and/or rhizomes, and their plant residues inhibited the germination and growth of some other plant species. These knotweeds, which are non-mycorrhizal plants, also suppressed the abundance and species richness of arbuscular mycorrhizal fungi (AMF) in the rhizosphere soil. Such suppression was critical for most territorial plants to form the mutualism with AMF, which enhances the nutrient and water uptake, and the tolerance against pathogens and stress conditions. Several allelochemicals such as flavanols, stilbenes, and quinones were identified in the extracts, residues, and rhizosphere soil of the knotweeds. The accumulated evidence suggests that some of those allelochemicals in knotweeds may be released into the rhizosphere soil through the decomposition process of their plant parts, and the exudation from their rhizomes and roots. Those allelochemicals may inhibit the germination and growth of native plants, and suppress the mycorrhizal colonization of native plants, which provides the knotweeds with a competitive advantage, and interrupts the regeneration processes of native plants. Therefore, allelopathy of knotweeds may contribute to establishing their new habitats in the introduced ranges as invasive plant species. It is the first review article focusing on the allelopathy of knotweeds.

## 1. Introduction

The genus *Fallopia* (Polygonaceae) contains highly competitive invasive species such as, Japanese knotweed (*Fallopia* *japonica* (Houtt.) Ronse Decraene; synonym: *Polygonum cuspidatum* Sieb. et Zucc.), giant knotweed (*Fallopia sachalinensis* (F. Schmidt) Ronse Decraene; synonym: *Polygonum sachalinensis* F. Schmidt ex Maxim), Bohemian knotweed (*Fallopia* x *bohemica* (Chrteket Chrtková) J.P. Bailey; synonym *Polygonum* x *bohemicum* (Chrtek et Chrtková) P.F. Zika et A.L. Jacobson). Japanese knotweed and giant knotweed hybridize naturally by sexual reproduction and create the hybrid Bohemian knotweed [1].

These knotweeds are fast-growing perennial herbaceous plants and form monospecific stands with dense canopy, and expand the stands through their extensive rhizome systems [2]. Japanese knotweed grows 2–3 m in height, and has multiple branches with hollow stems. Its shoots were recorded to grow 4–15 cm per day [3]. Its ovate leaves are 3–10 cm in length with a truncate base. Giant knotweed is 3–6 m in height, and has few branches with hollow stems. Its ovate leaves are 20–40 cm in length with a deeply cordate base. Characteristic of Bohemian knotweed has variations between both species [2].

Japanese knotweed grows under a wide range of soil pH, and nutrient poor soil conditions such as volcanic slopes as a pioneer species, and in disturbed areas such as roadsides and pastures [4,5,6]. Giant knotweed occurs in riparian corridors, coastal cliffs, road banks, and bare soils in human settlements [3]. Those knotweeds show a strong preference for man-made disturbed habitats, and along roads, and watercourses are their most frequent habitats. However, Bohemian knotweed has the highest population in man-made habitats [7,8].

Knotweeds spread sexually and asexually. The seeds of knotweeds are buoyant, and carried with water flow in streams and rivers for a long distance. The seeds are also easy to disperse with the wind [9,10]. The seed distribution may contribute to establishing knotweed populations in new habitats. Knotweeds also propagate with their rhizome branches. The rhizomes grow quickly, and the apex of the rhizome branches develops into an aerial shoot and forms a new shoot clump [4]. Rhizome fragments made by such as floods and human activities, as small as 1 cm in length and 0.7 g fresh in weight can regenerate new plants [11]. The fragmentation and subsequent regeneration increase their propagation potential.

The native range of Japanese knotweed is China, Japan, Korea, and Taiwan, and giant knotweed is native to Sakhalin Island, North Japan, and Korea [2]. Both species were introduced into Europe in the 19th century as ornamental plants and collections for botanical gardens, and were sold to gardens and parks in various countries [2,12]. Japanese knotweed was also cultivated as a Chinese medicinal plant [13]. Giant knotweed was recommended as riverbank stabilizer and livestock feed [14]. Although hybrid species, Bohemian knotweed was first recorded in Europe around 1980s, it was considered to occur in the late 19th century and spread undetectably [15]. The Japanese knotweed and giant knotweed were not sympatric in the native range. However, Bohemian knotweed was also observed in Japan in 1997 [16]. Japanese knotweed is the most widespread species in Europe, and followed by Bohemian knotweed and giant knotweed [8,17].

Japanese knotweed and giant knotweed were introduced into North America in the 19th century. The presence of Bohemian knotweed was first recognized in 2001 from Seattle, USA [18]. The presence of the knotweeds has been confirmed in 42 states including Alaska and 8 provinces in Canada [19,20]. These knotweeds were also confirmed in Australia, New Zealand, South Africa, and other countries [15,16]. Knotweed species have already naturalized in many countries as invasive noxious weeds, and are listed in the world’s 100 worst invasive alien species [21].

The characteristics of life-history of plants, such as high growth and high reproduction rate, and phenotypic plasticity are involved in the naturalization of invasive plants into the introduced range [22,23,24]. As described above, knotweeds grow fast and reproduce sexually and asexually. Those species can grow under poor soil conditions and in disturbed places. The single stem of Japanese knotweed bore about 200,000 seeds, and the germination rate was 50–80% [25,26]. Soil collected from the invaded site of Bohemian knotweed contained about 800 seeds/m^2^ [27]. Genetic diversity of each knotweed species is not clear. However, knotweeds showed large epigenetic differentiation and phenotypic plasticity in response to several environmental conditions [28,29]. Epigenetics diversity provides clonal plants with the potential for acclimation in various environmental conditions [30]. Hybridization of Japanese knotweed and giant knotweed probably gives genetic diversity to a hybrid plant, Bohemian knotweed [1,31,32].

High defense capacity against pathogens and herbivores contributes for the invasive plants to naturalize into introduced range [33,34,35]. Japanese knotweed has a lower abundance and diversity of invertebrate herbivores compared to native plant species of introduced range, and received less leaf damage than their native plant species [36]. Knotweed contains flavones, quinones, and stilbenes [13,37], and some of these compounds may act as defensive agents against herbivores and pathogens. In addition, aqueous extracts of rhizomes of Japanese knotweed altered soil fauna and reduced nematode population [38].

The interactions of the invasive plants with native plant communities are also crucial. Successful invasive plants often have allelopathic properties [39,40,41]. According to the literature, knotweeds probably release allelochemicals into their rhizosphere soil. Those allelochemicals may inhibit the germination and growth of neighboring plant species, and cause the reduction in seedling recruitment of the native plant species in the introduced range. However, there has been no review article focusing on the allelopathy of knotweeds. The objective of this review is to discuss the possible involvement of allelopathy in the invasiveness of knotweed. This paper presents an overview of the allelopathic property and allelochemicals of knotweeds, and a discussion of the importance of allelopathy for the knotweed invasion.

## 2. Allelopathy of Knotweeds

Allelopathy is the interaction between one plant and other neighboring plants through the specific secondary metabolites which are defined as allelochemicals [42]. The allelochemicals are released into their rhizosphere soil and neighboring environments either by root exudation, decomposition of plant residues, rainfall leachates, or volatilization from living plant parts [43,44,45].

Plant-to-plant interaction is a complex combination of competition for resources such as water, nutrients, and light, along with allelopathic interaction through allelochemicals [46,47]. Thus, it is essential to eliminate such competitive effects from experimental systems to clarify allelopathy [48,49,50]. Bohemian knotweed reduced the growth and survival rate of native plant species, *Eupatorium perfoliatum* L. and *Acer saccharinum* L. in field conditions. Supplemental nutrient and light to the growth conditions for those native plants recovered the inhibitory effects of the knotweed, but the recovery was limited. Therefore, allelopathy of Bohemian knotweed may contribute to a certain extent of the reduction in the growth and survival rate of the native plant species [51].

A rhizome (contain single node) of Bohemian knotweed and each of six native plant species (four forbs; *Geraniun robertianum* L., *Lamium maculatum* L., *Silene dioica* (L.) Clairv. and *Symphytum officinalis* L.; and two grasses; *Lolium perenne* L. and *Poa trivialis* L.) were grown together in 7 L pots filled with soil for three weeks. The rhizome of Bohemian knotweed significantly suppressed the growth of four forbs but not grasses. Activated carbons mixed with soil in the pots reduced the inhibitory effects of the knotweed on the forbs [37]. Activated carbon absorbs organic compounds in the soil [52]. Therefore, the result indicates that allelochemicals released from the rhizome of Bohemian knotweed may be involved in the inhibition. The evidence of allelopathy of knotweeds has been accumulated over three decades. In this section, allelopathic potential of the exudation, extracts, and plant residues of knotweeds was summarized (Table 1). 

### 2.1. Exudation

Japanese knotweed was grown in “donor pots”, and test plants (*Salix viminalis* L., *Salix atrocinerea* Brot. and *Populus nigra* L.) were grown in “target pots”. Test plants were irrigated with drain solution from the donor pots and with additional nutrient-enriched solution, and incubated for four months. The system could discriminate resource competition between Japanese knotweed and test plants. The growth of those test plants was suppressed by the drain solution from Japanese knotweed. The drain solution contained polyphenol compounds, but those compounds were not identified [53]. Giant knotweed (donor plant) and *Lactuca sativa* L. seedlings (receiver plant) were incubated with root exudate recirculating system [63] for 10–14 days. Exudate from roots and rhizomes of giant knotweed significantly inhibited the growth of *L. sativa* seedlings [54]. According to those observations, exudation from rhizome and/or roots of those knotweeds may contain allelochemicals, which cause the suppression of the growth of the test plant species. Although the organs that synthesize those allelochemicals are unknown, certain allelochemicals may be released from rhizomes and/or roots of knotweeds. Therefore, the evaluation of allelopathic potential of extracts of rhizome and/or roots is necessary.

Aqueous rhizome extracts of Japanese knotweed, giant knotweed, and Bohemian knotweed inhibited the root and hypocotyl growth of *Leucosinapis alba* (L.) Spach. However, the inhibitory effects of three extracts were not significantly different [55]. Aqueous rhizome extracts of Japanese knotweed and Bohemian knotweed also delayed the germination of *Raphanus sativus* L., and suppressed their growth. The roots of *R. sativus* showed symptoms of oxidative stress such as abnormal shapes of nuclei, plasma membrane, mitochondria, and endoplasmic reticulum [56,57]. Aqueous rhizome extracts of Japanese knotweed (*Polygonum cuspidatum* s.l.) inhibited the biomass of the mosses, *Atrichum angustatum* (Brid.) Bruch and Schimp. and *Thuidium delicatulum* (Hedw.) Schimp. [58]. Those findings indicate that the rhizomes of knotweeds may contain allelochemicals, and those allelochemicals are extractable.

### 2.2. Plant Residues

Japanese, giant, and bohemian knotweeds are perennial herbaceous plants, but above-ground parts of the knotweeds die back at the first frost in the winter season [21]. Those above-ground parts decay and accumulate as a litter layer on the soil. During the decomposition process of the litter, some of the secondary metabolites may be liberated into rhizosphere soil and act as allelochemicals [43,44,45]. Therefore, evaluation of the allelopathic potential of above-ground parts of knotweeds is also necessary.

Leaves of Japanese knotweed, giant knotweed, and Bohemian knotweed were shattered into small pieces and mixed with soil, and the seeds of *Leucosinapis alba* (L.) Spach, *Brassica napa* L., *Chenopodium album* L., and *Echinochloa crus-galli* (L.) P.Beauv were sown into the mixture. The germination of *L. alba* and *B. napa* was significantly suppressed by the residues of those knotweed leaf residues. The inhibitory activity of the residue of Japanese knotweed was the highest. However, all residues did not significantly inhibit the germination of *C. album* and *E. crus-galli* [59]. Aqueous leaf extracts of Japanese knotweed, giant knotweed, and Bohemian knotweed inhibited the germination of *Urtica dioica* L., *Calamagrostis epigejos* (L.) Roth, and *Lepidium sativum* L. However, the inhibitory effect of Japanese knotweed was the least [60]. Senescent above-ground parts of Japanese knotweed were soaked in water. Seeds of *Triticum aestivum* L. and *Sinapis arvensis* L. were sown into the soil, and irrigated with the soaking water of the Japanese knotweed every two days for two weeks. The irrigation of the soaking water resulted in significantly reduction in their germination [61]. Aqueous extracts of whole plants of Japanese knotweed and giant knotweed also inhibited the growth of *Brassica napa* L., *Avena sativa* L., and *Helianthus annuus* L. [62]. Those findings indicate that above-ground parts of those knotweeds may contain allelochemicals. Those compounds may be released into the soil during the decomposition process of the plant residues. However, the efficiency of allelopathic potential among Japanese knotweed, giant knotweed, and Bohemian knotweed is not able to compared because of the limited information.

## 3. Allelochemicals Found in Knotweeds

**Figure 1:**
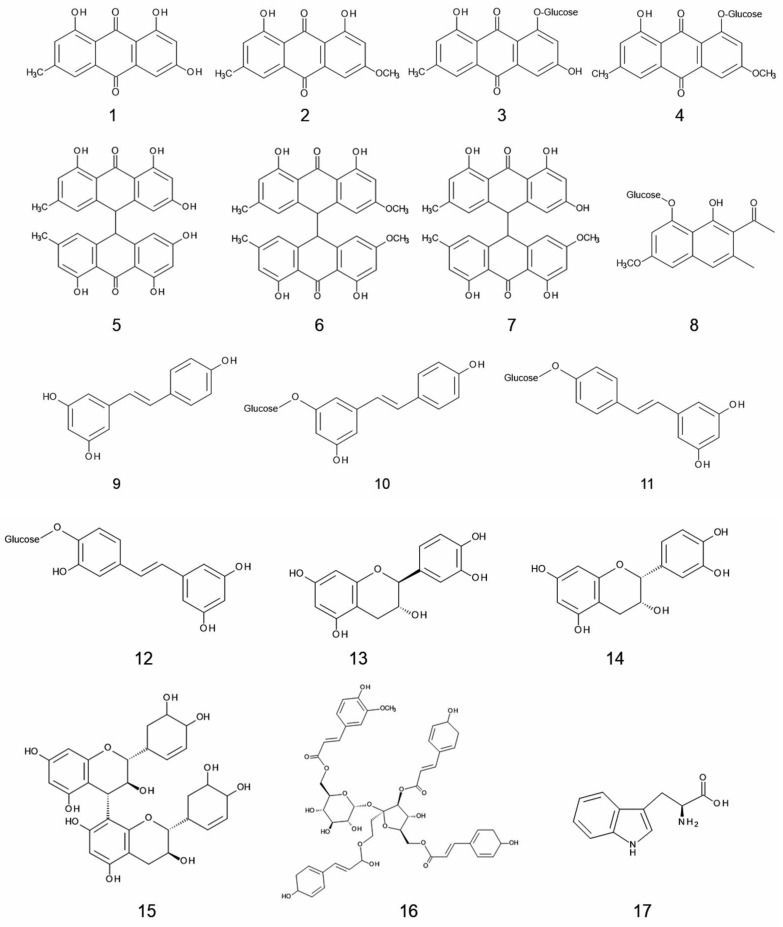
Allelochemicals identified in knotweeds. The numbering of chemical compounds is a continuation of the numbering given in Table 1.

Based on the observations described in the previous section, knotweeds may be allelopathic and release certain allelochemicals through the decomposition process of plant parts, and the exudation from their rhizomes and/or roots into their rhizosphere soil and neighboring environments. Therefore, identification of those allelochemicals is important to understand the allelopathy of knotweeds. Allelochemicals identified in knotweeds were shown in Table 2 and Figure 1.

Extracts of rhizomes and roots of giant knotweed inhibited the growth of *Lactuca sativa* L. seedlings, and two quinones, emodin (**1**) and physcion (**2**) were isolated and identified in the extracts. Emodin inhibited the growth of *L. sativa*, *Amaranth* spp. and *Phleum pratense* L. at concentrations greater than 50–100 mg L^−1^. However, physcion only inhibited *L. sativa* at 200 mg L^−1^. The concentrations of emodin in knotweed were 158, 72, and 213 mg kg^−1^ dry weight of its rhizomes, aerial parts, and fallen leaves, respectively, and 55 mg kg^−1^ dry weight of its rhizosphere soil. Those of physcion were 32, 22, and 180 mg kg^−1^ dry weight of the rhizomes, aerial parts, and fallen leaves, respectively, and 30 mg kg^−1^ dry weight of the soil [54]. Considering their inhibitory activity, the concentration of both compounds in the rhizosphere soil may be enough to cause the growth inhibition. In addition, those glycosides, emorin-1-*O*-β-D-glucoside (**3**) and physcion-1-*O*-β-D-glucoside (**4**) were isolated from the rhizomes and aerial parts of the knotweed [54]. Although both glucosides did not show any growth inhibitory activity, emodin and physcion may be liberated from those compounds during decomposition process of the plant parts of the knotweed.

Eleven compounds, emodin (**1**), physcion (**2**), emodin 8-*O*-glucoside (synonym; emorin-1-*O*-β-D-glucoside: (**3**), physcion-8-*O*-glucoside (synonym; physcion-1-*O*-β-D-glucoside: (**4**), piceid (**10**), resveratroloside (**11**), piceatannol glucoside (**12**), (-)-catechin (**13**), (-)-epicatechin (**14**), procyanidin B_3_ (**15**), and vanicoside B (**16**) were isolated and identified in Japanese knotweed roots [64]. Emodin, piceid, resveratroloside, piceatannol glucoside, (-)-catechin, (-)-epicatechin, and vanicoside B showed inhibitory activity against the growth of *Lactuca sativa* L. Among them, (-)-epicatechin has the highest growth inhibitory activity [64,67].

Resveratrol (**9**) was found in roots, leaves, stems, and flowers of Japanese knotweed and its concentration in the roots was the highest [68]. Emodin (**1**), resveratrol (**9**), and (-)-epicatechin (**14**) significantly suppressed the root growth of *Raphanus sativus* L., and (-)-epicatechin had the highest growth inhibitory activity among them. When solution of resveratrol (0.2 mg/mL), (-)-epicatechin (0.61 mg/mL), or emodin (0.2 mg/mL) was applied to *R. sativus* every 4 days during 14 days, resveratrol and emodin were detected at very low concentrations in the extract of *R. sativus*, while (-)-epicatechin was not detected in the extracts [69]. This result indicates that these compounds may be absorbed by the plants and cause growth inhibition. Moreover, these compounds may be metabolized in the plants, and exist only a small amount of the compounds in the plants.

Freeze-dried roots of Japanese knotweed, giant knotweed, and Bohemian knotweeds were extracted with aqueous methanol and centrifuged, and their supernatants were analyzed with LC-Mass spectrum. Thirteen compounds were identified in Japanese knotweed, 9 compounds for giant knotweeds, and 14 compounds for Bohemian knotweeds [66] (Table 2). Resveratrol (**9**), piceid (**10**), resveratroloside (**11**), catechin (**13**), and epicatechin (**14**) were also identified in the spring sprouts of Japanese knotweed, giant knotweed, and Bohemian knotweeds. Concentrations of resveratrol, piceid, resveratroloside, catechin and epicatechin in the sprouts was 64, 683, 48, 103, and 568 mg kg^−1^ dry weight for Japanese knotweed, 29, 502, 31, 167, and 674 mg kg^−1^ dry weight for giant knotweeds, and 23, 215, 11, 41, and 230 mg kg^−1^ dry weight for Bohemian knotweed, respectively. Therefore, the sprouts of Bohemian knotweed contained less amount of piceid, resveratroloside, catechin, and epicatechin compared to other sprouts [65]. Among those identified compounds in Table 2, L-tryptophan (**17**) was reported to have inhibitory activity against several plant species. The compound may be released into rhizosphere soil by the decomposition of plant litter and rainfall leachates [70,71]. Emodin (**1**) was reported to work as an allelopathic agent [54]. Resveratrol (**9**) and piceid (**10**) were also shown to have growth inhibitory activity on *Lepidium sativum* L. [67].

(-)-Catechin (**13**) and (-)-epicatechin (**14**) inhibited the growth of several other plant species [64,72]. Inhibitory activity of (-)-catechin was more active than (+)-catechin, and (-)-catechin was considered to act as an allelochemical for invasive plant species, *Centaurea stoebe* L. to succeed its invasion in North America. In their hypothesis [40,73], this compound may be released from the roots of *C. stoebe* into the soil and disturb the regeneration of the native plant species by the inhibition of their germination and growth. However, by far less amount of catechin was found in the field soil to cause growth inhibition of native plant species [74]. Therefore, it is necessary to determine the concentrations of identified allelochemicals of knotweeds in the rhizosphere soil, and to clarify the contribution of those identified allelochemicals to allelopathy of knotweeds. In addition, inhibitory activity of those compounds should be evaluated with plant species in the introduced range of knotweeds.

**Figure 2:**
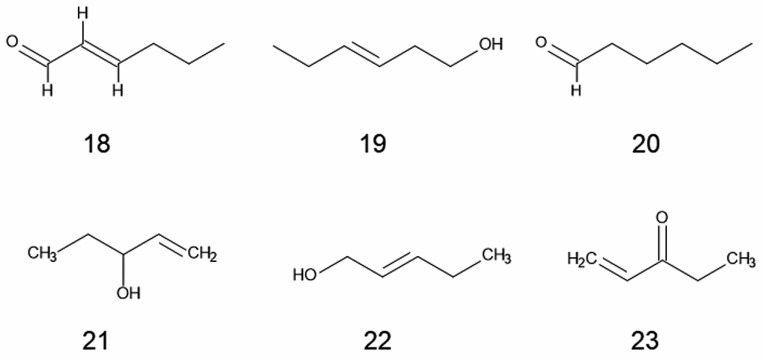
Volatile compounds found in the leaf extracts of Japanese knotweed.

Eighteen volatile compounds were found in the leaf extracts of Japanese knotweed. Main compounds in the extracts were 2-hexenal (73.4% of total compounds, Figure 2; **18**), 3-hexen-1-ol (7.0%, **19**), *n*-hexanal (2.8%, **20**), 1-penten-3-ol (2.6%, **21**), 2-penten-1-ol (2.2%, **22**), and ethyl vinyl ketone (1.1%, **23**) [75]. However, allelopathic activity of those compounds for knotweed invasion is not clear.

Japanese knotweed is a traditional Chinese medicinal herb. Its roots and rhizomes have been used for over 100 prescriptions in treatments for jaundice, inflammation, scald, favus, and hyperlipemia diseases [76]. Sixty-seven compounds including stilbenes, quinones, flavonoids, coumarins, and lignans have been identified in the roots and rhizomes of Japanese knotweed, and have been investigated in pharmacological activity, such as hepatoprotective, anti-inflammatory, estrogenic, anticancer, antiviral, antibacterial, and antifungal effects [13]. Although allelopathic and/or phytotoxic activity of most of those compounds have not been investigated, some of those compounds may possess phytotoxic activity. For example, quercetin inhibited the growth of several plant species, and it was reported to work as an allelopathic agent [77,78]. Protocatechuic acid was also shown to have phytotoxic activity [79].

## 4. Invasion and Allelopathy of Knotweeds

Perennial plants are able to release allelochemicals into the rhizosphere soil over several years through the decomposition process of plant parts including fallen leaves, and the exudation from their rhizomes and roots, and those allelochemicals may be able to accumulate in the soil [80,81,82,83,84,85,86]. The invasion of perennial herbaceous species, knotweed significantly reduced the plant diversity and abundance of native herbs, shrubs, and juvenile trees in the introduced range [87,88]. The invasion of knotweed also suppressed long-term native tree regeneration and shifted from tree-dominated riparian forests to knotweed-dominated herbaceous shrublands [89].

According to the novel weapon hypothesis, some invasive plant species may have particularly strong allelopathic activity and success in their invasion into the introduced range. Allelopathy of the invasive plants is mediated by allelochemicals that are new to the plant species in the introduced range. Those indigenous plants species in the introduced range are susceptible to the allelochemicals. The plants that co-evolved with invasive plants had the opportunity to obtain the defense systems against those allelochemicals. However, the plant species in the introduced range have not obtained the defense systems that obviate those allelochemicals [40,73]. Root powder of Japanese knotweed suppressed the germination of *Ulmus minor* Mill. (native in Europe), but did not suppress the germination of *Ulmus parvifolia* Jacq. (native in East Asia, same as knotweed) [90]. The concentrations of piceid (**10**) and resveratrolside (**11**) in Japanese knotweed from Switzerland (introduced range) were higher than in those from China (native range) [67]. Those observations may support the novel weapon hypothesis for the invasive plant species.

In addition, the invasion of Japanese knotweed reduced the abundance and species richness of arbuscular mycorrhizal fungi (AMF) [91]. Root powder of Japanese knotweed also inhibited AMF vesicle formation in *Ulmus* spp. [90]. Mycorrhizal colonization is crucial for most territorial plants. AMF fungi are widespread and important mycorrhiza for plant symbionts. AMF fungi increase the ability of plants to absorb nutrient and water, and enhance the protection potential against pathogen attacks and several stress conditions [92]. Knotweeds are non-mycorrhizal plants and do not form arbuscular mycorrhiza [93,94]. Knotweeds may be able to degrade the fungal mutualism of nearby plants without negatively impacting their own nutrient and water acquisition. The reduction in AMF populations in the soil of the knotweed introduced range weakens the ability of residential plants for the competition and regeneration, and leads knotweed to dominant communities in the introduced range. Allelochemicals have the potential to inhibit plant mutualism with AMF fungi [95]. Therefore, allelochemicals released from the knotweeds may cause the reduction in AMF communities in the soil, and contribute to the knotweed invasion. The suppression by allelochemicals on AMF communities were also found in non-mycorrhizal species, *Alliaria petiolate* (M. Bieb.) Cavara and Grande (Brassicaceae) [96,97].

Soaking water of giant knotweed (aboveground parts) suppressed the population of soil fungal pathogens [61]. Bohemian knotweed and indigenous plant species were grown in the soil collected from knotweed-uninvaded areas. The community of soil bacteria shifted the balance in favor of the knotweed, and promoted the growth and regeneration of the knotweed more than those of indigenous plant species. This promotion effect was reduced by adding activated carbons to the soil [31]. The observation suggests that certain compounds released from the knotweed may change the balance of soil bacteria in favor of the knotweed. However, further investigation is necessary to identify those allelochemicals.

**Figure 3:**
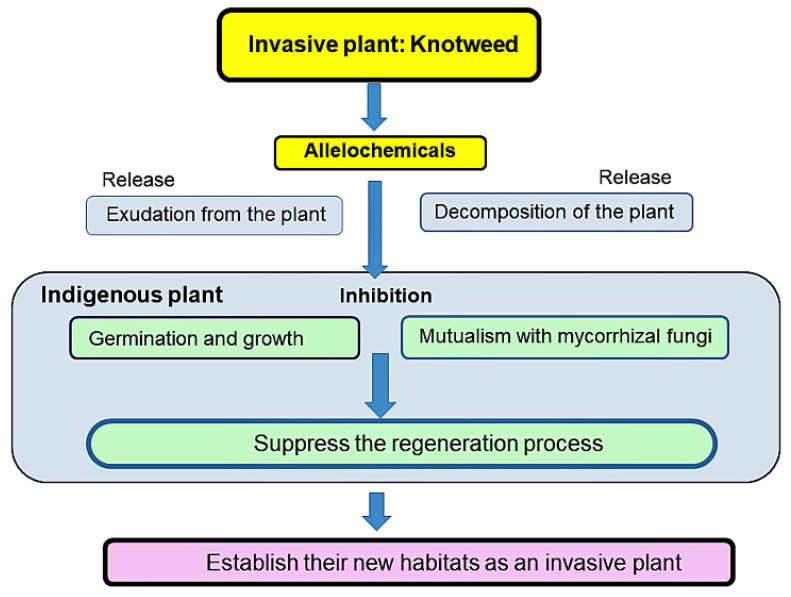
A possible scheme of knotweed to establish the new habitats.

Many of the phytotoxic substances from the invasive plants have been reported to have multiple functions such as allelopathic, anti-pathogen, anti-herbivore activity, and provide the invasive plants with the advantage in the increasing their population in the introduced range [24,41,98]. Large numbers of secondary metabolites have been isolated from knotweeds, such as stilbenes, quinones, flavonoids, coumarins, and lignans [13]. Among them, for instance, emodin was reported to work as a defense compound for pathogens, herbivores, and abiotic stress factors [99]. Resveratrol and piceid may also be involved in the interaction of plant–pathogen and plant–herbivore [100,101]. Japanese knotweed plant itself was also reported to possess antipathogen and antifungal effects [13,76]. Some of those compounds may enhance competitive ability of knotweeds and make the plant invasive. As describe previously, knotweeds may interrupt the regeneration process of indigenous plant species by decreasing their germination and growth directory, and indirectly via the suppression of mycorrhizal colonization to indigenous plant species (Figure 3). In addition, the elevated temperature from 2000 to 2008 results in an increase to 35–53% of the habitat being suitable for knotweed in southern Ontario, Canada [102], indicating that global warming may increase the threat of the invasion of the species into the additional introduced areas.

## 5. Conclusions

Japanese, giant, and Bohemian knotweeds are invasive and often form dense monospecies stands through the interruption of the regeneration process of indigenous plant species by suppressing their germination and growth. The evidence summarized in this paper indicates that those knotweeds have allelopathic properties (Table 1), and contain several allelochemicals (Table 2; Figure 1 and Figure 2). Some of those allelochemicals may be released into the rhizosphere soil and neighboring environments through the decomposition process of plant parts of the knotweeds and the exudation from their rhizomes and roots. Those released allelochemicals can suppress the germination and growth of indigenous plant species, and may also cause the reduction in AMF communities in the soil. Mycorrhizal colonization is crucial for most territorial plants because AMF fungi enhance the nutrient and water uptake, and the tolerance against pathogen and stress conditions. Therefore, those allelochemicals released from knotweeds may provide the knotweeds with the competitive advantage against the indigenous plants, and interrupt the regeneration process of the indigenous plant species. Thus, allelopathy of knotweeds may contribute establishing their new habitats as invasive plant species. However, it is necessary to determine the specific activity of those identified allelochemicals on the indigenous plant species, and their concentration in the rhizosphere soil for the evaluation of the contribution of those allelochemicals to the allelopathy of knotweeds. It is also necessary to identify allelochemicals involved in the suppression of AMF colonization of indigenous plant species.

## Figures and Tables

**Table 1:** Allelopathic activities of roots, rhizomes, and above-ground parts of knotweeds.

Source		Knotweed	Target Plant Species	Inhibition	Reference
Root, rhizome					
	Exudation	Japanese knotweed	*Salix viminalis, Salix atrocinerea, Populus nigra*	Growth	[53]
		Giant knotweed	*Lactuca sativa*	Growth	[54]
	Rhizome extract	Japanese knotweed Giant knotweed Bohemian knotweed	*Leucosinapis alba*	Growth	[55]
		Japanese knotweed Bohemian knotweed	*Raphanus sativus*	Growth Germination	[56,57]
		Japanese knotweed	*Atrichum angustatum* *Thuidium delicatulum*	Biomass	[58]
Above-ground part					
	Leaf residue	Japanese knotweed Giant knotweed Bohemian knotweed	*Leucosinapis alba* *Brassica napa*	Germination	[59]
	Leaf extract	Japanese knotweed Giant knotweed Bohemian knotweed	*Urtica dioica* *Calamagrostis epigejos* *Lepidium sativum*	Germination	[60]
	Soaking water	Japanese knotweed	*Triticum aestivum* *Sinapis arvensis*	Germination	[61]
	Extract (whole part)	Japanese knotweed Giant knotweed	*Brassica napa, Avena sativa* *Helianthus annuus*	Growth	[62]

**Table 2:** Allelochemicals identified in knotweeds.

Reference		[64]	[54]	[65]			[66]		
Phytochemical Class	Compound	J	G	J	G	B	J	G	B
Quinone	Emodin (**1**)	✓	✓				✓	✓	✓
	Physcion (**2**)	✓	✓				✓	✓	✓
	Emorin-1-*O*-β-D-glucoside (**3**)	✓	✓						✓
	Physcion-1-*O*-β-D-glucoside (**4**)	✓	✓						
	Emodin dianthrone (**5**)						✓	✓	✓
	Fallopion (**6**)						✓	✓	✓
	Physcion dianthron (**7**)						✓	✓	✓
	Torachrysone glucoside (**8**)						✓	✓	✓
Stilbene	Resvertrol (**9**)						✓		✓
	Piceid (**10**)	✓		✓	✓	✓	✓		✓
	Resveratroloside (**11**)	✓		✓	✓	✓	✓		✓
	Piceatannol glucoside (**12**)	✓					✓		✓
Flavanoid	(-)-Catechin (**13**)	✓		✓	✓	✓	✓	✓	✓
	(-)-Epicatechin (**14**)	✓		✓	✓	✓	✓	✓	✓
	Procyanidin B_3_ (**15**)	✓							
Phenylpropanoid	Vanicoside B (**16**)	✓		✓	✓	✓			
Indole	Tryptphan (**17**)						✓	✓	✓

Japanese knotweed (J), Giant knotweed (G), Bohemia knotweed (B).
